# Neurodevelopmental outcome of late preterm infants in Johannesburg, South Africa

**DOI:** 10.1186/s12887-018-1296-3

**Published:** 2018-10-15

**Authors:** Tanusha Ramdin, Daynia Ballot, David Rakotsoane, Lethile Madzudzo, Nicolette Brown, Tobias Chirwa, Peter Cooper, Victor Davies

**Affiliations:** 1 Neonatal Unit, Department of Paediatrics and Child Health, Charlotte Maxeke Johannesburg Academic Hospital, School of Clinical Medicine, University of the Witwatersrand, Johannesburg, South Africa; 2 Department of Biostatistics, School of Public Health, University of the Witwatersrand, Johannesburg, South Africa

**Keywords:** Infant, Premature. Child development, Follow-up studies, South Africa, Developmental disabilities

## Abstract

**Background:**

Late preterm infants, previously considered low risk, have been identified to be at risk of developmental problems in infancy and early childhood. There is limited information on the outcome of these infants in low and middle income countries.

**Methods:**

Bayley scales of infant and toddler development, version III, were done on a group of late preterm infants in Johannesburg, South Africa. The mean composite cognitive, language and motor sub-scales were compared to those obtained from a group of typically developed control infants. Infants were considered to be “at risk” if the composite subscale score was below 85 and “disabled” if the composite subscale score was below 70. Infants identified with cerebral palsy were also reported.

**Results:**

56 of 73 (76.7%) late preterm infants enrolled in the study had at least one Bayley assessment at a mean age of 16.5 months (95% CI 15.2–17.6). The mean birth weight was 1.9 kg (95%CI 1.8–2.0) and mean gestational age 33.0 weeks (95% CI 32.56–33.51). There was no difference in the mean cognitive subscales between late preterm infants and controls (95.4 9, 95% CI 91.2–99.5 vs 91.9.95% CI 87.7–96.0). There was similarly no difference in mean language subscales (94.5, 95% CI 91.3–97.7 vs 95.9, 95% CI 92.9–99.0) or motor subscales (96.2, 95% CI 91.8–100.7 vs 97.6, 95% CI 94.7–100.5). There were four late preterm infants who were classified as disabled, two of whom had cerebral palsy. None of the control group was disabled.

**Conclusions:**

This study demonstrates that overall developmental outcome, as assessed by the Bayley scales of infant and toddler development, was not different between late preterm infants and a group of normal controls. However, 7.1% of the late preterm infants, had evidence of developmental disability. Thus late preterm infants in low and middle income countries require long term follow up to monitor developmental outcome. In a resource limited setting, this may best be achieved by including a parental screening questionnaire, such as the Ages and Stages Questionnaire, in the routine well baby clinic visits.

## Background

One in every three pre-school children in low and middle income countries (LMICS) globally fails to meet normal milestones in socio-emotional and cognitive spheres [1]. The majority of these infants were from sub-Saharan Africa. Preterm birth is a potential risk factor for later neurodevelopmental impairment. Until recently, late preterm infants born between 34 and 37 completed weeks, were considered to be at low risk of morbidity and developmental problems. There is, however, increasing evidence that late preterm infants (LPI) are at increased risk of neonatal problems and poor neurodevelopmental function, in comparison to their term counterparts [2–5]. The incidence of problems increases as gestational age decreases. Elective preterm delivery should therefore be discouraged, LPI should be discharged 48 h after birth and have appropriate long term follow up [2, 3, 5]. In South Africa, limited health resources necessitate measures to reduce the workload of doctors. For example, very low birth weight infants, who are at increased risk of complications, are discharged at earlier chronological ages and lower weights compared to well-resourced settings [6]. In the same context, apparently healthy neonates who are considered to be low risk, including late preterm infants (LPI), are not managed by medical staff but are routinely examined by midwives at birth, discharged to their mothers and followed up at the well-baby immunization clinics.

There is a lack of data from LMICS on the long term outcome of children who sustained insults in the neonatal period; this is especially problematic in sub-Saharan Africa [7]. In a middle income country, such as South Africa, improved health care has resulted in increased neonatal survival rates, but follow up data is lagging behind [7]. Reliable information on the burden of long term morbidity related to neonatal problems is therefore minimal [7].

There are three published systematic reviews of studies evaluating the neurodevelopmental outcome of late preterm infants [3, 5, 8]. All three reviews concluded that LPI are at increased risk of neurodevelopmental disability in comparison to their term counterparts and recommend closer follow up of these infants. Most of the studies were conducted in high income countries, particularly the United States of America. There were no studies from sub-Saharan Africa. The aim of this study was therefore to determine the neurodevelopmental outcome of late preterm infants in Johannesburg, South Africa in comparison to a group of term control infants.

## Methods

This was a prospective follow up study conducted in the neonatal unit of a tertiary hospital in Johannesburg, South Africa. Late preterm infants (LPI) were defined as those infants with a birth weight above 1500 g and a gestational age below 37 weeks. LPI who were born between 1 July 2013 and 30 June 2014 and had required admission at birth, who were discharged from the neonatal unit were invited to attend the study clinic.

The study group comprised those LPI infants who had attended at least one follow-up study clinic visit. Infants with congenital abnormalities likely to affect neurodevelopment, in particular Trisomy 21, were excluded from the study. A group of well term babies who had gone home with their mothers after birth during the same period were enrolled as a control group and followed up at the same clinic. The developmental outcome of the control group has been reported elsewhere [9].

Gestational age was assessed by maternal menstrual history and clinical assessment using the Ballard score [10, 11]. Infants were classified as appropriate for gestational age (AGA) or small for gestational age (SGA) using the Fenton Growth calculator for preterm infants (https://peditools.org/fenton2013/).

Children were seen at the study clinic every three months. Developmental assessment was done using the Bayley scales of infant and toddler development, version III (BSITD III). It was anticipated that a large number of children would drop out of the follow up study, so BSITD (III) assessments were done at 9 to 12 months and then again at 15 to 18 months of age. If a child defaulted, the BSITD (III) assessment would be done at the next visit. The BSITD (III) assessments for both the study and control participants were done by an appropriately trained physiotherapist or paediatrician. A Cronbach’s alpha intra-class correlation of 0.89 was determined between different observers. The assessor was blinded to the participant’s neonatal history, including the gestational age. The BSITD (III) scores were calculated using the age corrected for prematurity. In order to ensure a reasonable rate of follow up, a text message was sent to remind parents of the appointment, transport costs were refunded and defaulting patients were traced and rebooked. If developmental problems were identified, the child was referred for appropriate intervention. The child’s weight, height and skull circumference were measured at each visit and plotted on World Health Organization (http://www.who.int/childgrowth/standards/chart_catalogue/en/) growth charts; the growth parameters were expressed as Z scores derived from these charts.

### Sample size calculation

A previous study conducted in the same unit, found the mean composite cognitive score in a group of very low birth weight infants to be 89 with a standard deviation of 15 [12]. Assuming the mean of the control group to be 97, a sample size of 44 would be required with α = 0.05 and β = 0.80, to detect a significant difference between the means [9]. Therefore a sample size of 50 participants was calculated for this study.

### Statistical analysis

Data was entered managed using Research Electronic Data Capture (REDCap ™) software, hosted by the University of the Witwatersrand [13]. Data was exported into IBM SPSS 23 for statistical analysis. The latest BSITD (III) score for each child was used for the analysis. The composite cognitive, language and motor scores were used as outcome variables. Continuous variables were normally distributed, so data was described using mean and 95% confidence intervals (95% CI). A group of 50 typically developed control participants from the same unit [9] who had been tested at the same mean adjusted age were used as controls. The mean composite cognitive, language and motor subscales were compared between the study participants and controls using unpaired t tests. A *p* value of 0.05 was considered to be significant.

Developmental delay was classified “at risk” if a composite BSITD (III) score was below 85 on any of the language, cognitive or motor scales and as “delayed” if a composite BSITD (III) score was below 70 on any of the sub-scales [12]. Cerebral palsy was diagnosed if there was a delay in motor milestones together with abnormal movement and/or posture [14]. The differences in the number of study participants with “at risk” or delayed development as compared to controls, were investigated using Chi Square or Fisher’s exact test. Univariate analysis using binary logistic regression was used to establish maternal and neonatal variables associated with a score below 85 on each of the BSITD (III) subscales. Maternal variables included demographic, obstetric, educational level and socio-economic status and neonatal variables included demographic, birthweight, gestational age, neonatal morbidity and growth parameters. Variables with a significant association at *p* < 0.1 were entered into a multivariable logistic regression model to determine adjusted odds ratios for significant determinants of a BSITD (III) score below 85 on each sub-scale.

## Results

**Fig. 1:**
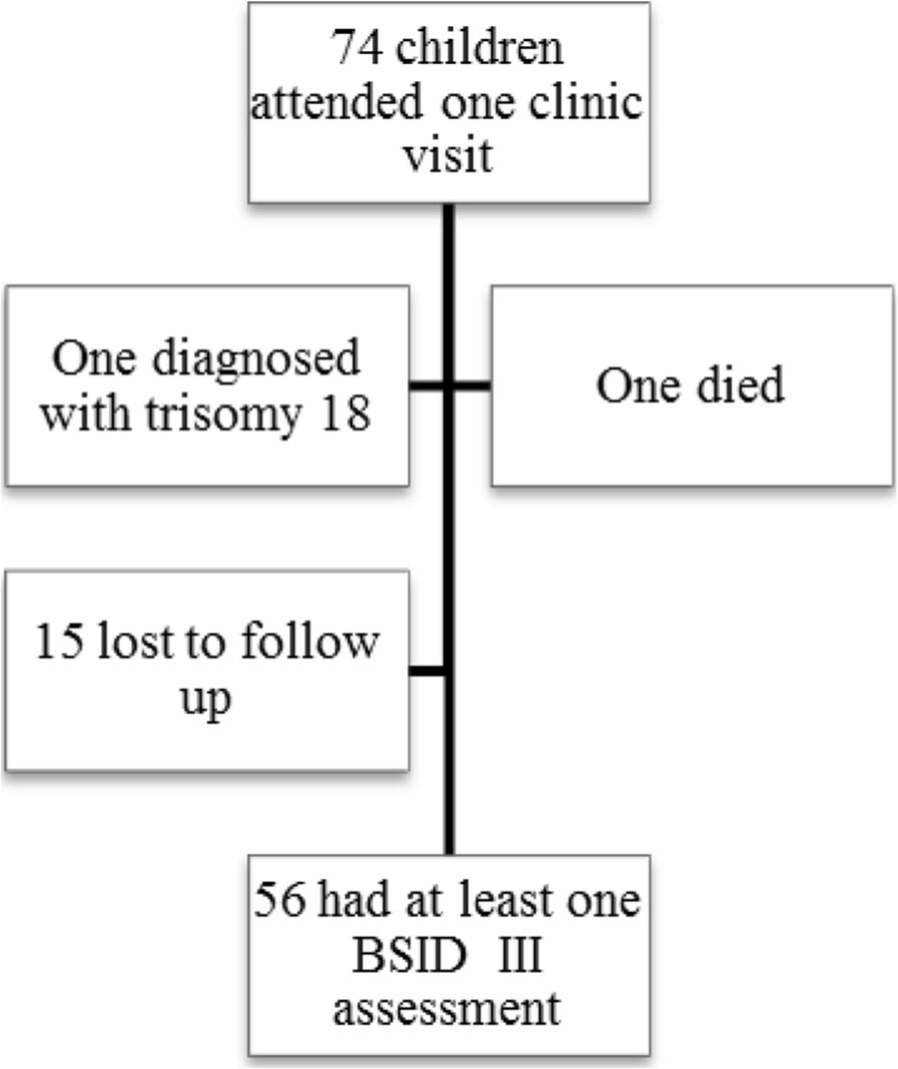
Derivation of final sample

A total of 73 LPI attended at least one neonatal follow up clinic. One child was diagnosed with trisomy 18 and was excluded. One child died and a further 15 defaulted from follow up. There were thus 56 infants in the final sample, corresponding to a follow up rate of 76.7% (56/73). (see Fig. 1).

The mean birth weight was 1.9 kg (95%CI 1.8–2.0) and mean gestational age 33.0 weeks (95% CI 32.56–33.51). The majority of infants (54; 96.4%) were black African. There were no babies with early onset sepsis. Other demographic, maternal and neonatal clinical characteristics are shown in Table 1.

**Table 1:** Demographic and clinical characteristics of late preterm infants

Characteristic	Frequency	Percentage
Female	35	62.5
Appropriate for gestational age	49	87.5
Inborn	52	92.9
Primparous mother	16	28.6
Antenatal care	50	89.3
Antenatal steroids	3	5.4
Antenatal magnesium sulphate	2	3.6
Maternal HIV	24	42.9
Caesarean section delivery	31	55.4
Multiple gestation	14	25
Resuscitated in the delivery room	17	30.4
Severe IVH (grade 3 or 4)	2	3.6
Respiratory distress syndrome	26	46.4
Nasal CPAP	21	37.5
Mechanical ventilation	8	14.3
Postnatal steroids	1	1.8
Necrotising enterocolitis (stage 2 or 3)	3	5.4
Exchange transfusion	1	1.8
Late onset sepsis	9	16.1
Breastfed on discharge	27	48.2

The control group of infants has been described elsewhere. [9] The mean composite cognitive, language and motor subscales compared to the control group are shown in Table 2. The mean age of assessment in the study group was 16.5 months (95% CI 15.2–17.6).

**Table 2:** Comparison of mean composite scores for Bayley III subscales between late preterm infants and controls

Subscale	Study infants		Control infants		*P* values
	Mean	95% CI	Mean	95% CI	
Cognitive	95.4	91.2–99.5	91.9	87.7–96.0	0.24
Language	94.5	91.3–97.7	95.9	92.9–99.0	0.51
Motor	96.2	91.8–100.7	97.6	94.7–100.5	0.50

The number of LPI whose neurodevelopment was classified as “at risk” or disabled are shown in Table 3.

**Table 3:** At risk and disabled late preterm infants compared to controls

Subscale	At Risk		*P* value	Disabled	
	Study	Controls		Study	Controls
Motor	9 (16.1%)	3 (6.0%)	0.13	4 (7.1%)	0
Language	7 (12.5%)	4 (8.0%)	0.5	2 (3.6%)	0
Cognitive	14 (25.0%)	11 (22.0%)	0.82	2 (3.6%)	0

There were two study participants diagnosed with cerebral palsy – one was disabled in cognitive, motor and language functions, whereas the second had associated cognitive disability alone. The child with global disability had surgery for jejunal atresia with a complicated neonatal course, including sepsis and prolonged ventilation.

None of the demographic, maternal or neonatal factors was significantly associated with at risk neurodevelopmental status.

## Discussion

This is the first study to report on the neurodevelopmental outcome of LPI in sub-Saharan Africa. There is little reliable long term follow up data of high risk neonates in LMICS [7]. This information is essential to understanding the burden of disability in this context in order to inform health budgets and policies to ensure proper care for these children. Neonatal care and its related complications in LMICS are likely to be very different to those in high income countries, due to a variety of factors, including a different disease profile and limited health resources [7].

The present study did not demonstrate any statistically significant difference in the overall performance of LPI in comparison to typically developed control infants in the BSITD (III) assessment. The mean cognitive, language and motor scores were not different. However, there were four LPI who were classified as disabled, two of whom had cerebral palsy. This corresponds to a disability rate of 7% in this group of LPI, indicating that this group of infants warrants long term follow up.

The results of the current study are in agreement with other research, who report that LPI are at increased risk of neurodevelopmental disability in comparison to term infants [3, 5, 8]. In a large population based study, Johnson et al. found that LPI were at twice the risk of neurodevelopmental disability, primarily in the cognitive domain [4]. Researchers in Thailand and China also found developmental delay at the age of 12 months in LPI [15, 16]. The hospital admission policy during the study period stated that only LPI with neonatal problems would be attended by a paediatrician and admitted to the neonatal unit. The developmental outcome of those LPI who were not admitted is unknown. The results from the current study may therefore be an over-representation of developmental problems in this group.

The cause of neurodevelopmental delay in LPI appears may be caused by impaired brain development related to preterm delivery. Magnetic resonance imaging of LPI confirms that these infants have smaller brain size, more immature gyral folding and less developed myelination in comparison to infants born at term [17].

There is some suggestion that delayed development in LPI improves with chronological age. A report from Canada showed that parental reports of delayed development using the Ages and Stages Questionnaire (ASQ) decreased after one year of age, suggesting that the development of LPI catches up with other children over time [18]. The ASQ is a simple parent reported initial developmental screening instrument. The ASQ looks at personal, social, motor, problem solving, and communication for children from 2 to 66 months. This questionnaire can be completed in 12–18 min. It is cost effective and has been validated in different cultures and communities around the world [19]. The ASQ accurately identifies children who are in need of further evaluation and early intervention services. Most low risk LPI have Intelligence quotients (IQs) within the normal range at preschool age [20]. Infants who are delivered close to term (35 to 36 weeks gestation) still demonstrate developmental impairment [2]. Baron et al. present a strong case for avoiding elective preterm deliveries stating “Gestation is a developmental continuum best not interrupted during its natural course” [2].

The current study did not find any association between developmental status and neonatal or obstetric factors. Other reports have found male sex, maternal pre-eclampsia, low socio-economic status, emergency Caesarean section delivery and lack of breastfeeding on discharge to be associated with worse developmental outcome [4, 21].

Some researchers have found that LPI who demonstrate abnormal developmental outcome at their chronological age have appropriate development if their corrected age is used [22, 23]. This suggests that BSITD (III) should be done at corrected age [4]. In the present study, BSITD (III) assessments were done at the corrected age.

### Limitations of the study

The composite BSITD (III) scores were the primary end points of the study and were used to calculate the sample size. The study failed to demonstrate a significant difference in the rates of disability between LPI and term controls as the sample size was too small for this. The rate of disability in the LPI was 7% as opposed to none in the control group – indicating that the LPI are an at risk population.

Antenatal ultrasounds are not routinely performed at clinics. Gestational age of LPI was determined by last menstrual period or by Ballard scores.

The short follow up period made it difficult to confirm the presence and severity of cerebral palsy. Loss to follow up is an important limitation. Although the follow up rate of 76.7% in the present study is acceptable, it is possible that some of the defaulters were also disabled. Mothers may not see the point of bringing typically developed children back to repeated follow up, but stigma and emotional stress may result in decreased rates of follow up in disabled children [7].

The current study only included late preterm infants who had been admitted to the neonatal units according to protocol guidelines, including birthweight of less than 1800 g, respiratory distress, feeding problems or hypoglycaemia. The developmental outcome of those apparently healthy late preterm infants discharged to their mothers at birth remains unknown, because they were not admitted to the neonatal unit, and therefore were not followed up.

## Conclusion

The current study is the first report of developmental outcome in LPI in sub-Saharan Africa, and found a rate of disability of 7% in these infants. These findings are in agreement with reports from high income settings and confirm that LPI are an at risk population which requires close long term follow up, including neurodevelopmental. In a resource limited setting, this may best be achieved by including a parental questionnaire, such as the Ages and Stages Questionnaire, in the routine well baby clinic visits [24].

## Abbreviations

BSITD (III): Bayley scales of infant development, version III
LMICS: Low and middle income countries
LPI: Late preterm infants
REDCAP: Research Electronic Data Capture
